# A construct with fluorescent indicators for conditional expression of miRNA

**DOI:** 10.1186/1472-6750-8-77

**Published:** 2008-10-07

**Authors:** Linghua Qiu, Hongyan Wang, Xugang Xia, Hongxia Zhou, Zuoshang Xu

**Affiliations:** 1 Department of Biochemistry and Molecular Pharmacology, University of Massachusetts Medical School, 364 Plantation St, Worcester, MA 01605, USA; 2 Department of Pathology, Anatomy & Cell Biology, Thomas Jefferson University Medical College, 508 JAH, 1020 Locust Avenue, Philadelphia, PA 19107, USA; 3 Cell Biology, University of Massachusetts Medical School, 364 Plantation St, Worcester, MA 01605, USA; 4 Neuroscience Program, University of Massachusetts Medical School, 364 Plantation St, Worcester, MA 01605, USA

## Abstract

**Background:**

Transgenic RNAi holds promise as a simple, low-cost, and fast method for reverse genetics in mammals. It may be particularly useful for producing animal models for hypomorphic gene function. Inducible RNAi that permits spatially and temporally controllable gene silencing in vivo will enhance the power of transgenic RNAi approach. Furthermore, because microRNA (miRNA) targeting specific genes can be expressed simultaneously with protein coding genes, incorporation of fluorescent marker proteins can simplify the screening and analysis of transgenic RNAi animals.

**Results:**

We sought to optimally express a miRNA simultaneously with a fluorescent marker. We compared two construct designs. One expressed a red fluorescent protein (RFP) and a miRNA placed in its 3' untranslated region (UTR). The other expressed the same RFP and miRNA, but the precursor miRNA (pre-miRNA) coding sequence was placed in an intron that was inserted into the 3'-UTR. We found that the two constructs expressed comparable levels of miRNA. However, the intron-containing construct expressed a significantly higher level of RFP than the intron-less construct. Further experiments indicate that the 3'-UTR intron enhances RFP expression by its intrinsic gene-expression-enhancing activity and by eliminating the inhibitory effect of the pre-miRNA on the expression of RFP. Based on these findings, we incorporated the intron-embedded pre-miRNA design into a conditional expression construct that employed the Cre-loxP system. This construct initially expressed EGFP gene, which was flanked by loxP sites. After exposure to Cre recombinase, the transgene stopped EGFP expression and began expression of RFP and a miRNA, which silenced the expression of specific cellular genes.

**Conclusion:**

We have designed and tested a conditional miRNA-expression construct and showed that this construct expresses both the marker genes strongly and can silence the target gene efficiently upon Cre-mediated induction of the miRNA expression. This construct can be used to increase the efficiency of making cell lines or transgenic animals that stably express miRNA targeting specific genes.

## Background

RNAi is a conserved cellular mechanism that can mediate sequence-specific RNA degradation in eukaryotes [1] and has recently been harnessed for reverse genetics in mammalian cells and transgenic animals [2-5]. Many shRNA and miRNA transgene constructs have been designed and successfully used. The earliest type was composed of a Pol III promoter and a DNA sequence encoding a shRNA [6-10]. This type of constructs was constitutively active and was relatively inefficient in targeting the endogenous genes when pronuclear injection method was used to insert the transgene into the genome [7,11]. To overcome this limitation, several groups transfected cultured mouse embryonic stem cells, screened for clones where the transgene was active and then used those clones to produce transgenic mice [7,8,10]. Other groups used lentivirus to insert transgenes [12-14] or Pol II-promoter-based miRNA expression constructs, which drive the synthesis of a miRNA [15,16]. These approaches have achieved silencing of endogenous genes and specific loss-of-function phenotypes in transgenic mice.

To further improve the in vivo reverse genetics approach using RNAi, we need to overcome several shortcomings of the currently used transgene constructs. First, extremely high levels of shRNA expression should be avoided because such high levels of shRNA can induce non-specific toxicity [17]. The commonly used Pol III promoter U6 is a very strong and constitutively active promoter [18] that can drive shRNA expression to a very high level, which may lead to non-specific suppression of the endogenous miRNA expression [19,20]. By comparison, pol II promoters drive miRNA expression at lower levels and may be advantageous [4,21].

Second, although the constitutively active constructs work in cells and transgenic mice, a conditional silencing is more desirable. For example, if a target gene is essential, use of constitutively active constructs often yields no useful transgenic founders. This is because once injected into the embryo, the transgene immediately expresses the shRNA or miRNA, resulting in the silencing of the target gene and the death of the embryo. This problem can be avoided by using conditional constructs, which will not express the shRNA or miRNA when first injected, thus allowing the transgenic founders to be generated. Additionally, there is a need in biomedical research to understand hypomorphic phenotypes of a gene in a specific type of cells or at a specific time. A conditional construct will afford investigators this capability.

Third, coexpression of a reporter gene and a miRNA by one promoter can facilitate identification of miRNA expression in cells and in transgenic animals. One strategy for coexpression is to use a miRNA cassette inserted into the 3'UTR of a reporter gene, which has been explored by previous studies [22,23]. A shortcoming in this design is that the processing of primary miRNA (pri-miRNA) by Drosha will cause the mRNA to lose their 3' polyadenylation, which destabilizes the mRNA and reduces levels of the reporter gene expression. One solution to this problem is to embed the pre-miRNA in an intron as an intronic miRNA, which is common in many genomes [24]. Lin and colleagues [25] applied this strategy using an artificial in-frame intron placed within the open reading frame (ORF) of a reporter gene. This strategy is ingenious but relatively difficult to apply widely because one has to find sequences required for splicing (AG/GC with exon-splicing enhancers (ESEs) in its proximity) within the ORF, and this sequence requirement cannot always be met in an ORF.

In this report, we designed a new Pol II-based construct that overcomes these shortcomings. This construct uses a Pol II promoter to drive expression of the EGFP gene that is flanked by loxP sites. This feature will allow convenient screening for cells or transgenic mice where the transgene is active. Downstream to the EGFP gene is the gene encoding red fluorescent protein (RFP). In the 3' UTR of the RFP gene, we inserted an artificial intron containing a DNA sequence encoding a pre-miRNA. We show that this construct expresses EGFP under basal conditions. When induced by Cre recombinase, it drives expression of RFP and the miRNA that silences its target gene. We conclude that this construct may be used as a backbone for conditional gene silencing in transgenic mice.

## Results

### Finding efficient shRNAs against E1k and E2k subunits of KGDHC

**Figure 1:**
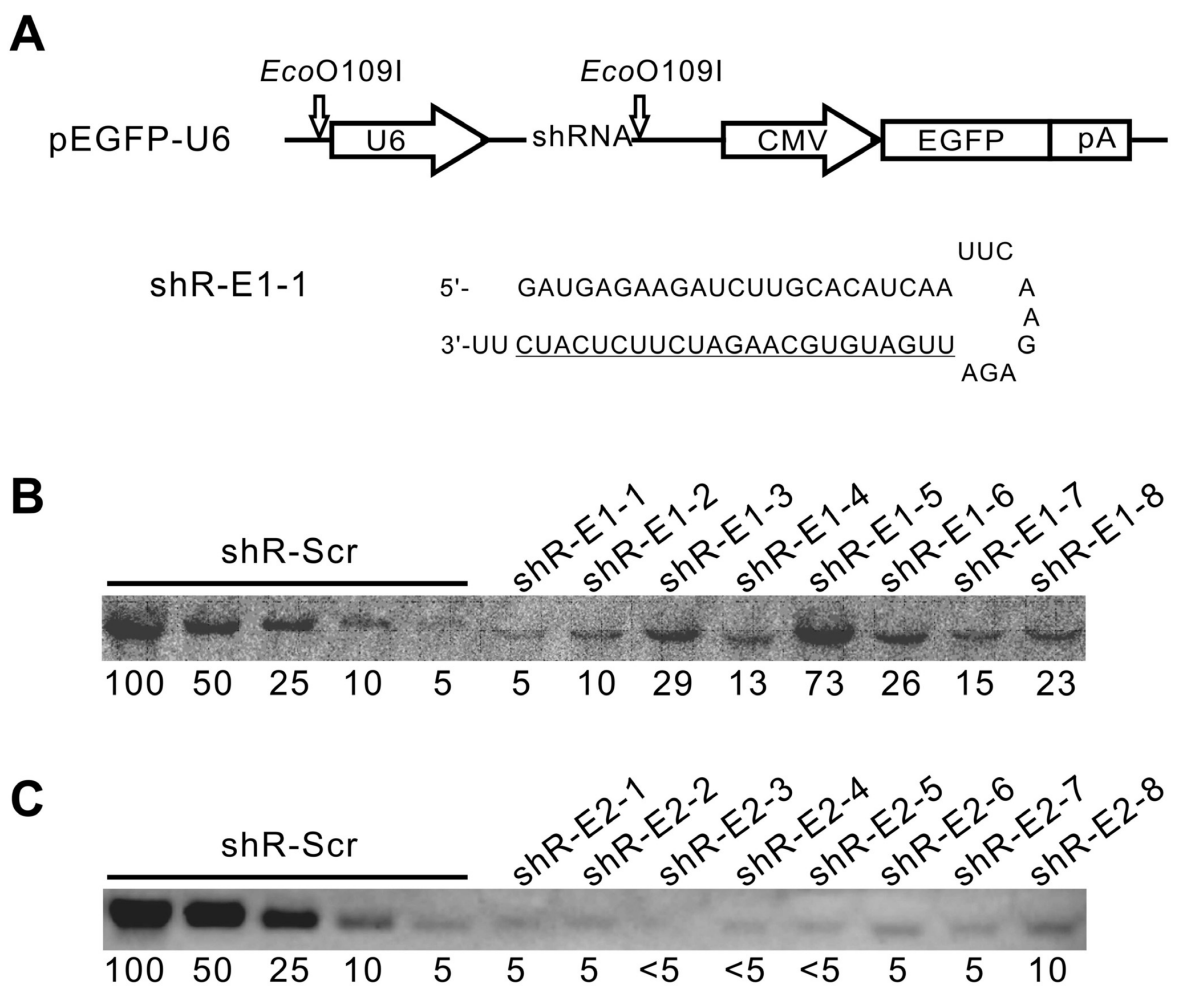
**Screen for shRNAs that silence E1k and E2k expression**. Eight shRNAs for each subunit were designed and constructed as described in Methods. (A) The structure of the shRNA-expressing constructs and an example for the shRNAs. (B) A Northern blot detecting silencing of the E1k subunit. Total RNA samples were prepared from cells 48 hours after transfection with the vectors shown in (A). Northern blot was carried out as described in Methods. The left five lanes are a dilution series of total RNA extracted from mouse NF1 cells transfected with a construct that expresses a scrambled shRNA (shR-Scr). The numbers at the bottom indicate the relative amount that was loaded in each lane. One hundred equals to 6 μg total RNA. In the right eight lanes, 6 μg total RNA extracted from cells transfected with each of the eight shRNAs against the E1k subunit were loaded. The probe detects the endogenous E1k mRNA. The numbers at the bottom indicate the relative density of the E1k band. (C) A Northern blot detecting silencing of the E2k subunit was done in the same way as in (B). The loading arrangement is the same as in (B) except that one hundred in the standard equals to 20 μg of total RNA. Twenty μg also is the amount of total RNA loaded in each sample lane.

We selected the E1k and E2k subunits of α-ketoglutarate dehydrogenase complex (KGDHC) as the targets for testing our miRNA-expressing constructs. KGDHC is a key enzyme in mitochondrial tricarboxylic acid (TCA) cycle and is essential for mitochondrial energy metabolism [26-29]. Dysfunction of KGDHC has been observed in the brains of Alzheimer's disease and could cause metabolic deficiency that is associated with the disease [30-35]. To build a miRNA expression construct that targets the E1k or the E2k subunit, our first step was to find shRNAs that can efficiently silence these two genes. We designed and constructed eight shRNAs targeting each of these two subunits. We used U6 promoter to drive the expression of these shRNAs (Figure 1A) and tested these constructs by transfecting them into mouse NF-1 cells. Forty eight hours after the transfection, we determined the mRNA levels of these two subunits using Northern blot. We used a serial dilution of RNA extracted from the cells transfected with a construct expressing scrambled shRNA as a standard (Figure 1B, C, shR-Scr lanes 1–5). This scrambled shRNA did not show any silencing activity towards these two genes (data not shown). By comparing to the standard, we found many excellent shRNAs that silenced the expression of E1k and E2k by more than 80% (Figure 1B, C).

### Placing miRNA in an intron does not affect the miRNA expression and target silencing but increases the reporter gene expression

**Figure 2:**
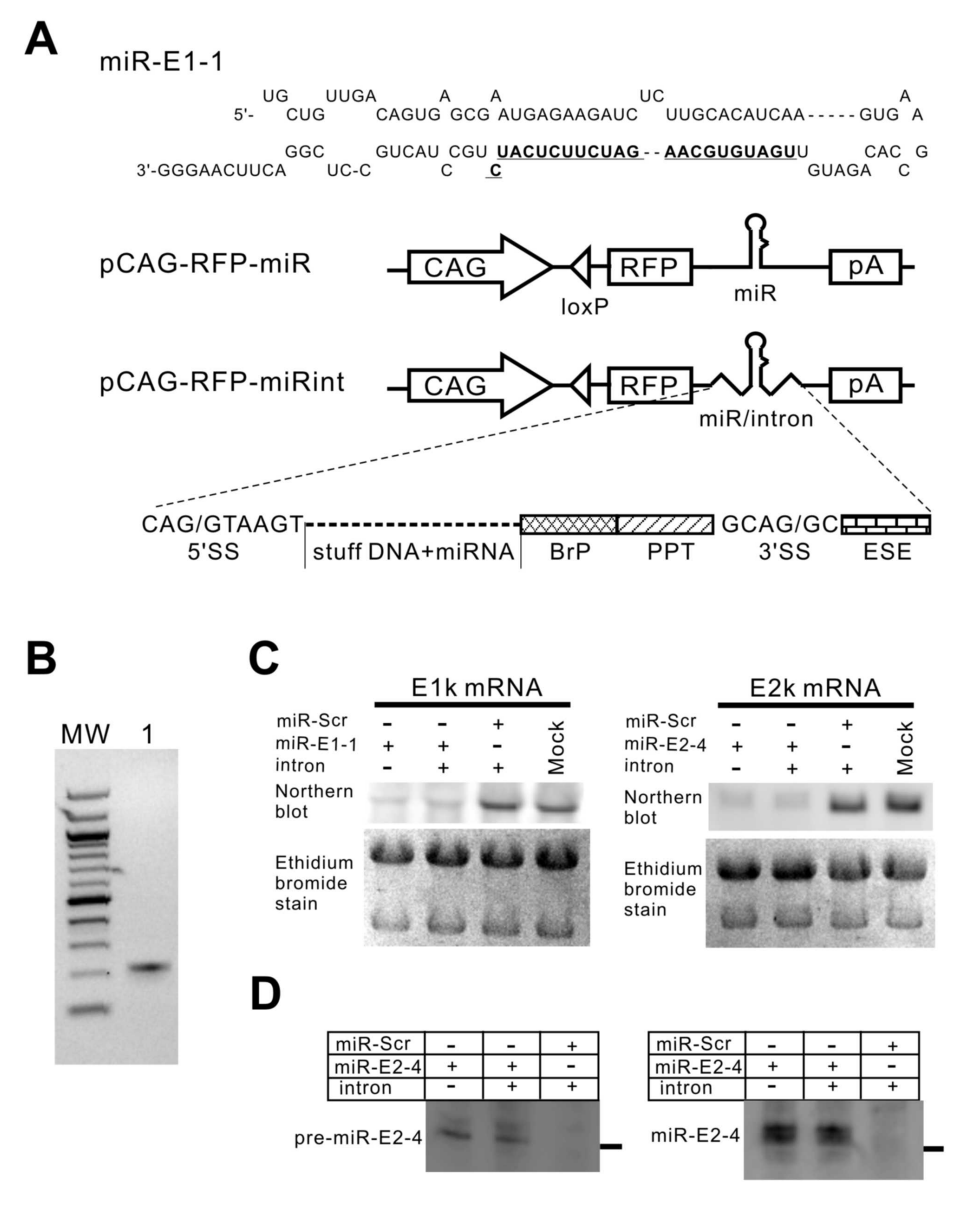
**Design and test of Pol II-driven miRNA-expression constructs**. (A) A schematic illustration of the construct design. From top to bottom: an example of miRNA against the E1k subunit (the bold, underlined sequence complements the E1k mRNA); construct pCAG-RFP-miR that constitutively expresses RFP and a miRNA, which is placed in the 3'-UTR of the RFP gene; construct pCAG-RFP-miRint that constitutively expresses RFP and a miRNA that is placed in an intron located in the 3'-UTR of the RFP gene; and structure of the artificial intron (SS = splicing site, BrP = branch point with the sequence TCCTGACCATTCAT, PPT = poly pyrimidine track with the sequence CCTCTTTCTTTTTCCT, and ESE = exon splicing enhancer with the sequence TTGTGGAAGAAAT). (B) Detection of intron-splicing in RNA extracted from NF1 cells that were transfected with pCAG-RFP-miR-E1-1int. The RT-PCR product was resolved on a 2% agarose gel (lane 1). MW (molecular weight) lane shows the 100 bp DNA ladder (New England Biolab). The PCR product was sequenced and the spliced exon junction sequence was ...TAATTAATTAACAG/GCTTGT..., which matched exactly as predicted from the correctly spliced junction. (C) Northern blots detecting E1k and E2k mRNAs. The RNA was extracted from cells 48 hours after transfection with the constructs pCAG-RFP-miR or pCAG-RFP-miRint. Ethidium bromide staining is shown as loading controls. (D) Northern blots detecting the pre-miR-E2-4 and miR-E2-4. The constructs pCAG-RFP-miR and pCAG-RFP-miRint were used in the transfection. The mark on the right side of the pre-miR-E2-4 gel indicates the position of a 60 nt single stranded DNA. The mark on the right side of the miR-E2-4 gel indicates the position of a 22 nt single stranded DNA.

We selected one effective hairpin against each of these two subunits (shR-E1-1, shR-E2-4) and incorporated the stem sequences into the pre-miR-30a structure to make miR-E1-1 and miR-E2-4, respectively (Figure 2A; also see [36]). We placed the pre-miR-E1-1 or pre-miR-E2-4 coding sequence behind a potent Pol II promoter CAG and in the 3'UTR of the RFP gene (Figure 2A). The CAG promoter is a ubiquitously active and commonly used promoter for gene expression in cells and in mice [37-40]. To mark the cells that express the miRNA, the CAG promoter also expresses RFP (pCAG-RFP-miR, Figure 2A). When transfected into cultured cells, these two constructs expressed RFP and knocked down their respective mRNAs for E1k and E2k to a similar degree as their shRNA counterparts (data not shown).

Many natural miRNAs are in introns of protein-encoding genes [24]. To determine whether placing a miRNA in an intron can improve the expression of the miRNA and the marker gene, we constructed an artificial intron and placed the pre-miRNA in this intron (pCAG-RFP-miRint, Figure 2A; also see methods). We placed this intron 10 nt-down stream from the stop codon because introns within the 50 nt downstream from the stop codon are found in some eukaryotic mRNAs and do not cause non-sense mediated decay (NMD) [41]. To test whether this intron can be successfully spliced, we extracted RNA from the cells transfected with pCAG-RFP-miRint and carried out RT-PCR across the splicing junction. We found a single band with the size of 212 bps that was expected from the spliced mRNA (Figure 2B). We did not detect any band with the size of 701 bps that would have been derived from the unspliced mRNA (Figure 2B). We sequenced the 212-bp PCR product and confirmed that the intron splicing occurred exactly as expected (see legend of Figure 2B). Thus, the artificial intron functions as designed. To compare pCAG-RFP-miRint with pCAG-RFP-miR, we transfected both constructs into the NF1 cells and determined their knockdown of target genes. By Northern blots, we detected no difference between these two constructs in their degree of knockdown of E1k and E2k mRNAs (Figure 2C), suggesting that intron placement of pre-miRNA encoding sequence does not increase the miRNA production. To confirm this, the pre-miR-E2-4 and miR-E2-4 levels were measured and the levels did not differ between the pCAG-RFP-miR and pCAG-RFP-miRint (Figure 2D). These results suggest that placing a pre-miRNA in an intron does not increase the miRNA expression.

**Figure 3:**
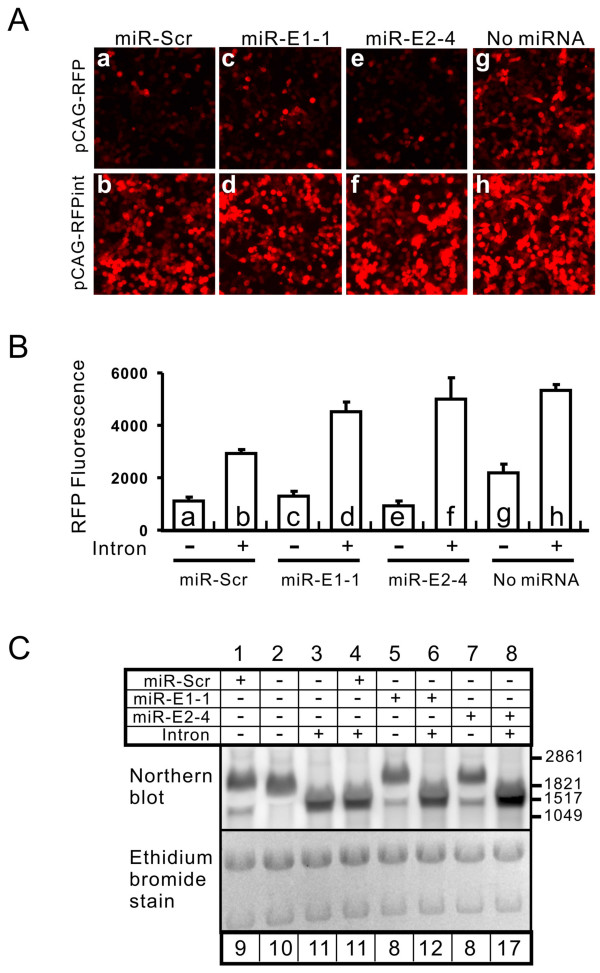
**Effects of pre-miRNA and intron on RFP expression**. (A) Representative photographs of cells that were transfected with various RFP-expressing constructs 48 hours before photographing. a, c and e were transfected with pCAG-RFP-miR constructs. b, d, f were transfected with pCAG-RFP-miRint constructs. g was transfected with pCAG-RFP, which does not contain a pre-miRNA. h was transfected with pCAG-RFPint, which has an intron but no pre-miRNA. (B) Quantification of RFP levels from the experiment represented in (A). (C) Northern blot detecting RFP mRNA synthesized by various pCAG-RFP constructs. The table on the top shows whether the construct contains miRNA or/and intron. The numbers in the bottom indicate the relative levels of mRNA (the thick band) that are normalized to lane 2, which is set at 10. Six μg of total RNA was loaded for each lane.

In order to determine whether the intron influences the expression of RFP, we compared the RFP expression after transfecting these constructs into the NF1 cells. The results revealed two roles of the intron. First, the intron enhanced the expression of RFP by more than twofold (compare panels g with h in Figure 3A, and bars g with h in Figure 3B, *p *< 0.01) despite an existing intron in the 5'UTR of the RFP gene in this construct. Second, a pre-miRNA in the 3' UTR of the RFP gene inhibited the RFP expression by 50% or more (compare panels a, c and e with panel g in Figure 3A, and bars with the same letters in Figure 3B, all *p *< 0.05). Placing the pre-miRNA in an intron, however, eliminated this inhibitory effect of the pre-miRNA (compare panels b, d and f with a, c and e, respectively in Figure 3A, and bars with the same letters in Figure 3B, all *p *< 0.01).

In order to establish whether the enhancing effect of the intron on RFP expression is a result of elevated mRNA levels, we examined RFP mRNA using Northern blot. Addition of intron increased mRNA levels by ~10% (compare lane 3 with lane 2 in Figure 3C). On the other hand, placing the pre-miRNA in the 3'-UTR of the RFP gene reduced the RFP mRNA by ~10–20% (compare lanes 1, 5 and 7 with lane 2 in Figure 3C) and resulted in a lower band (~1.5 kb, lanes 1, 5 and 7 in Figure 3C) that was consistent in size with the band expected from Drosha cropping. By placing the pre-miRNA in the intron, the decrease of mRNA levels caused by the presence of pre-miRNA in the 3'-UTR was largely prevented, and there was no lower band (lanes 4, 6 and 8 in Figure 3C). Overall, the changes in the mRNA levels caused by the addition of the intron did not fully account for the changes in RFP expression, suggesting that other post-transcriptional mechanisms also contribute to the increased levels of RFP expression.

**Figure 4:**
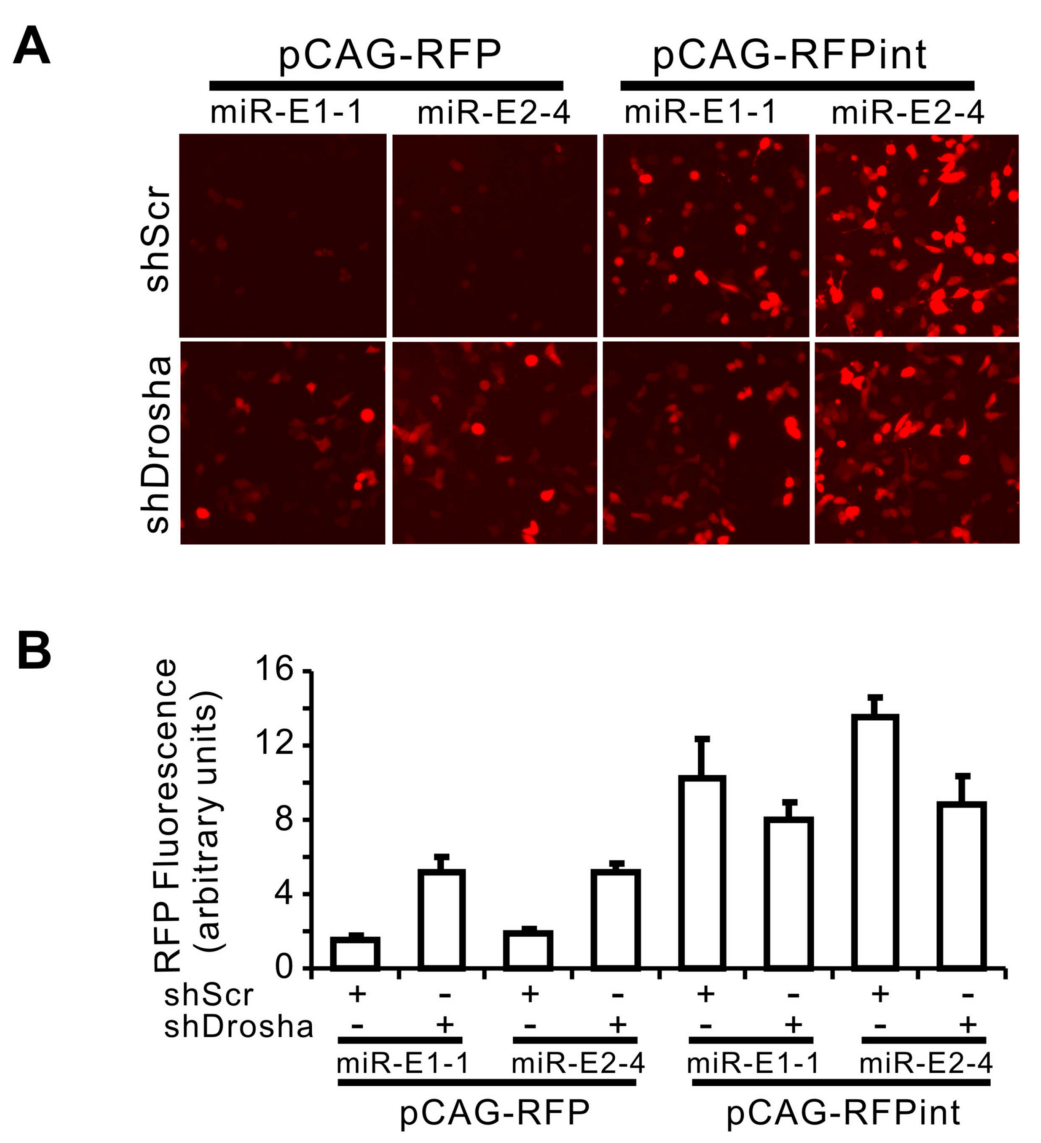
**Effects of Drosha on RFP expression**. Cells transfected with U6-shR-Drosha were cultured for 24 hours and then transfected with various pCAG-RFP constructs. After another 24 hours, RFP fluorescence was observed (A) and quantified (B).

To further determine the effect of primary miRNA (pri-miRNA) processing on the expression of miRNA, we silenced the expression of Drosha by transfecting the cells with a construct that expresses Drosha shRNA (U6-shR-Drosha) [16]. Silencing Drosha led to the recovery of RFP expression in constructs with the pre-miRNA placed directly in their 3'-UTR (see pCAG-RFP in Figure 4, *p *< 0.05 for both miR-E1-1 and miR-E-2-4) but lowered the RFP expression somewhat in constructs with the pre-miRNA embedded in an intron, although this did not reach statistical significance (see pCAG-RFPint in Figure 4). These results indicate that Drosha processing causes inhibition of RFP expression and placing the pre-miRNA in the intron makes the RFP expression independent from Drosha processing.

### An inducible construct with fluorescent indicators for expression of miRNA

**Figure 5:**
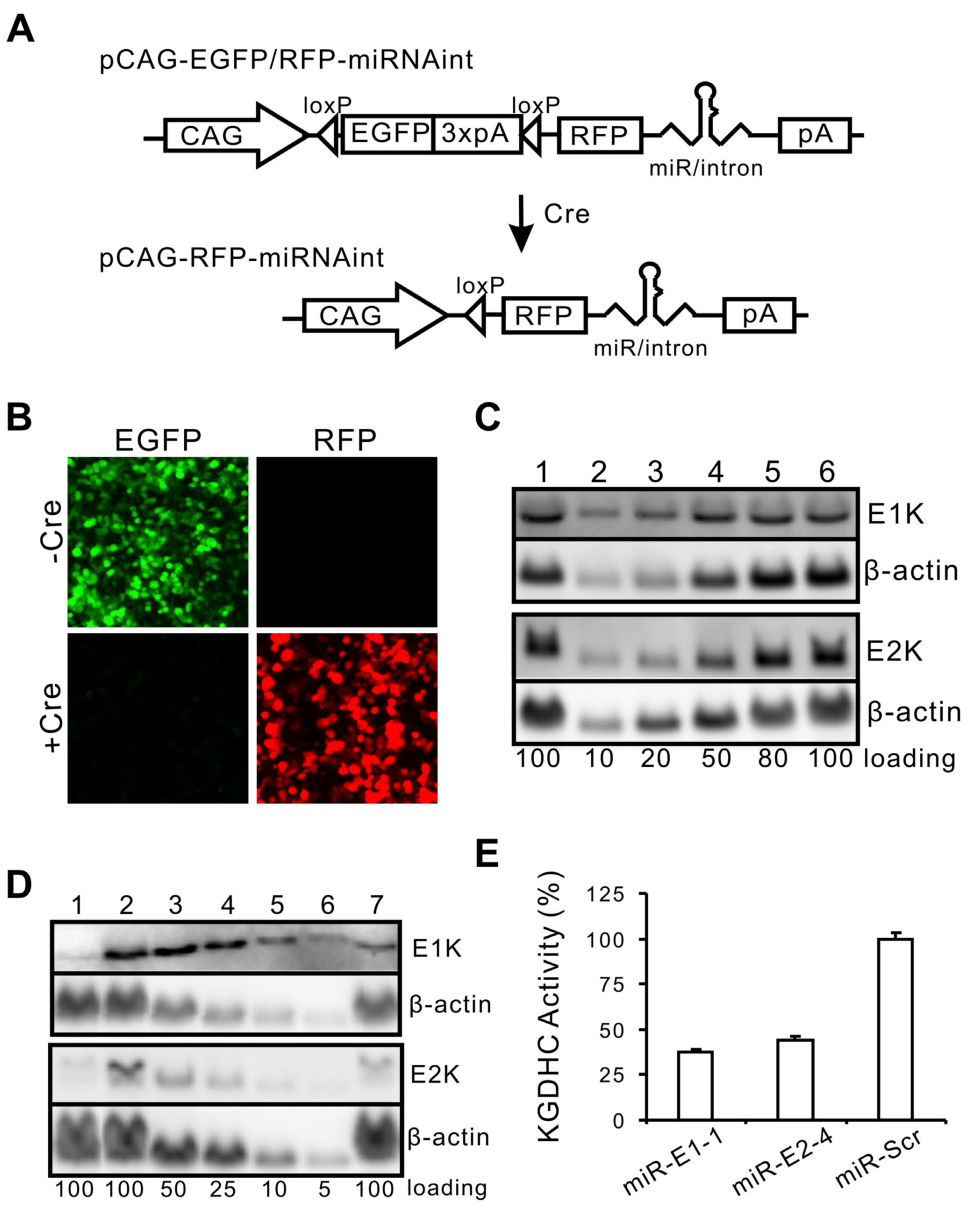
**Design and test of a Cre-inducible miRNA-expression construct with fluorescent markers**. (A) The basic construct of pCAG-EGFP/RFP-miRNAint is composed of the CAG promoter, a loxP site, the EGFP gene with 3 consecutive SV40 poly A signals (to ensure the cessation of the Pol II synthesis), a second loxP site, the RFP gene, a 3' UTR carrying the pre-miRNA hairpin, and a poly A signal. Cre mediates the excision of EGFP and the 3 poly A signals, allowing the CAG promoter to synthesize RFP and miRNA. (B) Top: after transient transfection with pCAG-EGFP/RFP-miRNAint and without Cre, EGFP was expressed and RFP was not. Bottom: cotransfection with Cre diminished EGFP expression and induced the RFP expression. (C) Northern blot of RNA extracted from cells transfected with pCAG-EGFP/RFP-miRNAint carrying miRNA against E1k or E2k but without Cre (lane 1). Lanes 2–6 are standard dilution of the RNA extracted from cells transfected with the scrambled miRNA construct. The relative RNA loadings are indicated at the bottom. One hundred represents 6 μg RNA for E1k blot (top) and 20 μg RNA for E2k blot (bottom). β-actin was detected as a loading control. (D) Northern blot of RNA extracted from cells cotransfected with pCAG-EGFP/RFP-miRNAint carrying miRNA against E1k or E2k and CMV-Cre (lane 7). Lane 1 is RNA from cells transfected with constitutively expressing pCAG-RFP-miRNAint constructs, which serve as positive knockdown control. Lanes 2–6 are standard dilution of the RNA extracted from cells transfected with the scrambled miRNA construct. The relative RNA loadings are indicated at the bottom. One hundred represent 6 μg RNA for E1k blot (top) and 20 μg RNA for E2k blot (bottom). β-actin was detected as a loading control. (E) Confirmation of protein knockdown by measurement of KGDHC activity. Three days after transfection of constructs CAG-RFP-miR-E1-1int, -miR-E2-4int or -miR-Scrint, NF-1 cells were lysed and assayed for KGDHC activity as described in Methods.

Having determined that intron-embedded pre-miRNA construct is superior, we incorporated this design in a conditional expression construct based on the Cre-loxP system (Figure 5A). The construct structure is based on the Z/AP construct that has been previously used in transgenic mice to express protein-encoding genes [42]. In the Z/AP construct, we replaced the reporter genes LacZ and human placental alkaline phosphatase gene, with EGFP and RFP, respectively. The resulting construct initially expresses EGFP gene, which is flanked by loxP sites. After induction with Cre, the EGFP gene is excised, and its expression ceases, leaving the CAG promoter to express RFP and the miRNA (Figure 5A). The EGFP fluorescence allows convenient monitoring of transgene expression in transfected cells or in vivo and can facilitate the subsequent analysis. We tested this construct by transfecting it with or without a Cre-expressing plasmid. Without Cre, only EGFP was expressed; with Cre, EGFP expression diminished but RFP expression appeared (Figure 5B). To further test whether the miRNA expression was regulated according to our design, we determined the target knockdown. We detected no knockdown of the mRNA targets without Cre (Figure 5C) and significant knockdown with Cre (Figure 5D). Thus, this construct worked as designed.

Finally, to determine whether this construct can knock down the target protein levels, we measured KGDHC activity in cells transfected with pCAG-RFP-miRNAint (Figure 5A). We observed a robust knockdown of the KGDHC activity by targeting either the E1k or the E2k subunit (both p < 0.01) (Figure 5E).

## Discussion

We have designed a conditional expression construct for miRNA expression, pCAG-EGFP/RFP-miRNAint (Figure 5A). This construct initially expresses EGFP. When exposed to Cre recombinase, the loxP-flanked EGFP gene is excised, allowing the CAG promoter to drive the expression of the miRNA and the marker RFP. This construct will be useful for generating conditional silencing in transgenic mice and will have several advantages compared with the currently available transgenic silencing constructs. For example, the constitutive Pol III or Pol II promoter-based constructs are inefficient in generating transgenic animals with high levels of knockdown [7,11]. Although, by screening for transfected ES cells with high levels of knockdown or using lentiviral delivery, transgenic mice with hypomorphic gene functions have been generated [10,12,13], these technologies are more complex and more costly than pronuclear injection. Furthermore, it will be difficult to obtain transgenic lines with a high degree of gene silencing if one targets an essential gene, because founders with high levels of shRNA or miRNA expression cannot survive. While this problem might be partially circumvented by expressing miRNA using a tissue-specific promoter [15], the conditional approach that we have designed here will be more versatile and a superior solution to this problem. By crossing with different Cre-expression lines, one can induce specific gene silencing in many different tissues from a single line of mice made with this conditional construct. Transgenic silencing mice can be made with conditional constructs using pronuclear injection method [2,43,44] or using more complex methods such as ES cell screening or lentiviral delivery. In addition, compared with the inducible transgenic RNAi constructs that have been reported in the literature, our conditional construct has the following advantages: It expresses EGFP before induction of the miRNA, thus affording a convenient screen for transgenic lines with desirable expression pattern. When crossed with a Cre driver transgenic line, the coexpression of RFP and miRNA will provide a convenient and precise indication as to in which cell type gene silencing is occurring.

While developing this construct, we compared the effects of placing the pre-miRNA in an intron with the effects of placing the pre-miRNA directly in the 3' UTR of the RFP gene. Our results indicate that placing the pre-miRNA in an intron does not increase the miRNA expression (Figure 2) but enhances the marker protein RFP expression (Figure 3). This conclusion agrees with a previous report, where a pre-miRNA was placed in a 5' intron in the EGFP gene [45]. Our analysis, however, further revealed that the intron enhances the RFP gene expression by two effects. First, intron can directly enhance the protein gene expression (compare bar h with bar g in Figure 3). Second, the pre-miRNA sequence placed in the 3'-UTR exerts an inhibitory effect on the protein gene expression, and this effect can be removed with the elimination of the pre-miRNA sequence by the intron splicing (Figure 3). These findings justify the inclusion of the intron in our conditional miRNA expression construct.

How the pre-miRNA placed in the 3'-UTR inhibits the RFP gene expression and why intron placement of the pre-miRNA eliminates this inhibition remain unknown. One model, as has been proposed [45], suggests that cropping of the pre-miRNA from the 3'-UTR by Drosha and DGCR8 complex led to a cleavage in the pri-miRNA, resulting in a separation between the 5'-Cap and 3'-poly A and the degradation of the residue mRNA, thereby reducing the RFP expression. By placing the pre-miRNA in an intron, the cropping of the pre-miRNA might occur in the spliced intron lariat. Alternatively, the cropping of pre-miRNA might occur before splicing, but this might not affect splicing [46]. In either case, the integrity of the mRNA will be protected, thus enhancing the RFP expression.

Changes in the RFP expression are consistent with the above models. When the pre-miRNA was present in the 3'-UTR, the RFP expression was decreased (compare bars a, c and e with bar g in Figure 3B) and this decrease was prevented by inhibition of Drosha (Figure 4); when the pre-miRNA was placed in the intron, the RFP expression was enhanced (compare bars a, c and e with bars b, d, and f, respectively) and inhibition of Drosha did not affect the RFP expression (Figure 4). However, at the mRNA level, the presence of pre-miRNA in the 3'-UTR caused only a slight decrease (compare lanes 1, 5 and 7 with lane 2 in Figure 3C); and placing the pre-miRNA in an intron caused a relatively small increase (compare lanes 1, 5 and 7 with lanes 4, 6 and 8). These changes are not sufficient to account for the changes in the RFP expression, thus suggesting that protection of mRNA from Drosha/DGCR8 processing is not the only mechanism whereby the intron placement of the pre-miRNA increases the RFP expression. From our data, it is clear that most of the enhancing effect of RFP expression is derived from the intrinsic properties of the intron, rather than protecting the mRNA from Drosha/DGCR8 cropping.

Our data also show that the presence of a miRNA in an intron does not interfere with intron splicing because the level of mature mRNA produced from the construct with intron alone was not higher than those from the constructs with intron containing pre-miRNA (Figure 3C, compare lane 3 with lanes 4, 6 and 8). Conversely, intron containing the pre-miRNA does not affect the miRNA processing because constructs with or without an intron produced similar levels of miRNA (Figure 2C). Thus, intron splicing and miRNA processing do not interfere with each other in a miRNA-containing intron. This conclusion agrees with a previous study [46]. However, our data also revealed that some pre-miRNA can dampen the intron-enhanced RFP expression (e.g. compare bar b with bar h in Figure 3B), possibly by interfering with the translation-enhancing effect of the intron. This observation indicates that the levels of the reporter gene expression cannot be used to compare the expression levels of different miRNA because the miRNAs may affect the reporter gene expression differently. Nevertheless, the reporter expression levels may be used as an indicator of the same miRNA expression levels in different cell populations, provided that the miRNA processing machinery is not saturated.

Based on our analysis, several improvements in our construct design is worth noting. First, we use a single promoter to drive both the reporter and the miRNA expression. This is advantageous compared with a commonly used design in Pol III-driven shRNA constructs, which places a reporter expression cassette on the same linear sequence with the shRNA expression cassette [12,13]. Because the synthesis of the reporter and the shRNA are driven by independent promoters, the reporter expression does not necessarily represent the shRNA expression and the degree of silencing. This is especially worrisome in transgenic animals, where a genomic insertion locus may influence different promoters differently. Our construct will not have this problem because the expression of the reporter and the miRNA is driven by a single promoter. Second, the intron-placement of pre-miRNA enhances the reporter expression, which improves from constructs that place the miRNA directly in the 3' UTR of the reporter [22,23] (Figure 3). Third, our placement of the intron in the 3' UTR makes our construct easily adaptable if one desires to use other reporters. The RFP in our construct can be easily replaced by any other commonly used reporter. This is advantageous compared with the construct using an in-frame intron within the open reading frame [25], where the reporter cannot be easily replaced.

## Conclusion

We have built a conditional miRNA-expression construct that expresses EGFP initially, and upon induction by Cre recombinase, expresses RFP and a miRNA, which mediates efficient silencing of the target gene. This construct can be used to increase the efficiency of making stable cell lines or transgenic animals that stably express miRNA for silencing specific genes.

## Methods

### Plasmid vectors

The pEGFP-U6 short hairpin expression vector (Figure 1A) was constructed by inserting the U6 promoter derived from BSENU6 [47] into pEGFP-N1 (Clontech) at the EcoO109I site (all restriction enzymes were obtained from New England Biolabs unless indicated otherwise). The U6 promoter fragment was amplified by PCR with introduction of EcoO109I sites at both ends using a pair of PCR primers (Forward: 5'-CAAGGCCCTTTGACGTCAATGGGAGTTTGTTTTGG-3'; Reverse: 5'-TTAGGGCCCGAGCGGATAACAATTTCACACAGGAA-3'). Additionally, the multiple cloning site (MCS) in pEGFP-N1 was modified by deleting the fragment between Hind III and Xma I. A shRNA coding sequence was cloned between the restriction enzyme sites PmeI and EcoRI downstream of the U6 promoter.

shRNAs against the E1k and E2k subunits of KGDHC were designed based on the asymmetry rule [48] as well as other rules [49]. The shRNA sequences were further screened by RNAfold analysis , which predicts minimum energy for secondary structures and pair probabilities, to exclude sequences with Tm of intrastrand folds greater than 55°C and by Blast homology searching to select sequences with minimal similarity with other mouse genes. The loop sequence (TTCAAGAGA) and the terminal poly-thymidines (TTTTT) were used for all shRNAs. The sense and antisense strands of shRNAs contain 23 nt. All the sense strands start with a "G" while the antisense strands complement the target mRNA sequences (Figure 1A). To insert shRNAs into the shRNA expression vector pEGFP-U6, two strands of synthetic DNA oligonucletides were annealed and cloned into the vector using the restriction sites PmeI and EcoRI. For each gene target, a total of eight shRNA plasmids were constructed. All constructs were verified by DNA sequencing. Target sequences for E1k are: shR-E1-1, GATGAGAAGATCTTGCACATCAA; shR-E1-2, GCAAGAACTAGCTTTGACGAGAT; shR-E1-3, GTTTCAACAGATTCGGTGCTATT; shR-E1-4, GCACAACCTAACGTCGACAAACT; shR-E1-5, GATGATGCTCCGGTAACTGTTTC; shR-E1-6, GATCTGGTGTGTTATCGACGAAA; shR-E1-7, GCAATTAGGACGTTTCAACAGAT; and shR-E1-8, GCATCGTATATGAGACCTTCCAT. Target sequences for E2k are: shR-E2-1, GTGCAATGCTGACGACTTTCAAT; shR-E2-2, GCAATGCTGACGACTTTCAATGA; shR-E2-3, GAGGTTGACATGAGTAACATACA; shR-E2-4, GGAAGTGGTGTATAGAGATTATA; shR-E2-5, GATATTGAACGGACCATTAATGA; shR-E2-6, GCAGCAGTAGAAGATCCAAGAGT; shR-E2-7, GAGGTTGACATGAGTAACATACA; and shR-E2-8, GCAGATATTGAACGGACCATTAA. The scrambled sequence is shR-Scr, GCGATGCTCTAAGGTTCTATCAA.

For miRNA expression, vectors based on CAG promoter were constructed. The lacZ and the human placental alkaline phosphatase (AP) genes in the pZ/AP vector [42] were replaced with EGFP, synthesized by PCR from pEGFP-N1 (Clontech), and RFP, synthesized by PCR from pDsRed2-C1 (Clontech), respectively. One cryptic start codon at the 5'UTR of the original AP gene that was not in frame was eliminated. These modifications produced a conditional vector for miRNA expression, pCAG-EGFP/RFP. A synthetic artificial intron (Figure 2A) was designed to contain typical intron splicing regulatory elements [50-52]. This intron was placed 10 nt-downstream of the stop codon of the RFP gene to avoid NMD [41]. A fragment of ~400 bases surrounding the miRNA insertion site was amplified from the first intron of the vector pUbC-miRNA-EGFP [36] with primers 5'-GCAATGACGCGATCGCTAATGCGGGAAAGCTCTTATTCGGGT-3' (forward) and 5'-AAGGCATGACGCGTTGTTGCGGCCGCCAGAGGTCCGGCGCCTGT-3' (reverse). The amplified fragment was digested using AsiSI and MluI and inserted into the artificial intron as stuff DNA, which can carry a miRNA (Figure 2A). This generated construct pCAG-EGFP/RFP-miRNAint (Figure 5A). By in vitro Cre-mediated excision, EGFP gene along with its poly A was eliminated to generate pCAG-RFP-miRNAint (Figure 5A), which expressed RFP and the miRNA constitutively.

miRNAs targeting E1k and E2k were derived from the shRNA sequences and were designed based on the pre-miR-30a structure (Figure 2A). Each strand of a miRNA was chemically synthesized. Two strands were annealed and cloned into the vector pUbC-EGFP as previously described [36]. The miRNA and its flanking sequences of about 200 bases at both sides from the vector pUbC-miRNA-EGFP were amplified by PCR and cloned to generate the pCAG-EGFP/RFP-miRNAint or pCAG-RFP-miRNAint vectors as described above.

The intron-less control vector pCAG-RFP-miRNA (Figure 2A) was constructed by amplifying the same miRNA and its flanking sequences from pUbC-miRNA-EGFP plasmid with SanDI site at both ends. After digestion with SanDI (Stratagene), the fragment was inserted directly into the 3'-UTR of the RFP gene at the SanDI site. The vectors U6-shR-Drosha and U6-shR-Scr with a shRNA targeting Drosha and a scrambled shRNA, respectively, were described previously [16].

### Cell culture and transfection

Mouse NF-1 cells were maintained in DMEM supplemented with 10% fetal bovine serum (Gibco). Cells were inoculated the day before transfection, which was performed at ~60% cell confluence using Lipofectamine 2000 (Invitrogen) based on the manufacturer's instructions. For pEGFP-U6-shRNA constructs and the constitutive miRNA expression constructs with CAG promoter, 4 μg DNA was used for each well in a 6-well plate. For the conditional miRNA constructs under the control of CAG promoter, 2 μg pCAG-EGFP/RFP-miRNAint and 6 μg CMV-Cre plasmids were cotransfected. In all experiments where multiple plasmids were used, the total plasmid amount was kept constant by supplementing with the empty vector.

### Fluorescence analysis

Cell images with fluorescence were taken using Zeiss Axiovert S100 microscope equipped with Axiocam camera and appropriate filters and were processed by Openlab software. For fluorescence quantification, total cell lysates were extracted using 1× reporter lysis buffer (Promega) with sonication and subsequent centrifugation. Protein concentrations were determined with BCA protein assay kit (Pierce) according to the manufacturer's instructions. The fluorescence intensity was determined using Tecan Safire microplate reader with appropriate excitation and emission wavelength for EGFP and DsRed2.

### Northern blots

Northern blot for mRNA was described previously [53] and used to determine the target knockdown. Briefly, two days following transfection, cells were harvested and total RNA was extracted using STAT-60 total RNA isolation reagent (Tel-Test). All mouse cDNA clones (Clone ID for E1K: 6417392; clone ID for E2k: 3583936) for probe preparation were purchased from Open Biosystems. Their cDNA sequences were verified by DNA sequencing. DIG-labeled probes were synthesized using the PCR DIG probe synthesis kit (Roche) according to the protocols of the manufacturer. The probe for RFP mRNA was derived from pDsRed2-C1 by PCR using primers 5'-GCCTCCTCCGAGAACGTCATCAC-3' (forward) and 5'-TAATACGACTCA CTATAGGGTGTAGTCCTCGTTGTGGGAGGT-3' (reverse). The E1k probe was derived from E1k cDNA using primers 5'-CACAGTCCCTGGTGGAAGCACA-3' (forward) and 5'-CCATCCGAGGGTCTGTGGTGAAG-3' (reverse). The E2k probe was derived from E2k cDNA using primers 5'-GACCAGGTTACCCTGACAACAGGA-3' (forward) and 5'-GCTGATGGTGAAGGTACCACCATC-3' (reverse). Dig-labeled RNA size marker was obtained from Roche. Northern images were visualized with the LAS-3000 imaging system and quantified by the Multi-Gauge software (FujiFilm).

For small RNA detection, RNA samples were prepared using mirVANA miRNA Isolation Kit (Ambion). Equal amounts of small RNA (1.5 μg) were resolved by 15% Acrylamide Sequagel (National Diagnostics). The RNA was transferred to a Nylon membrane (Ambion) with a semi-dry electroblotter (Owl Scientific). Dig-labeled RNA probes were synthesized with SP6/T7 Transcription Kit (Roche). Template DNA was derived from pCAG-RFP-miR-E1-1int and pCAG-RFP-miR-E2-4int by PCR with primers 5'-GAT CGCTAATGCGGGAAAGCTCTTATTC-3' (forward) and 5'-TAATACGACTCACTATAG GGAAAAAGAAAGAGGATGAATGGTCAG G-3' (reverse). The probe was approximately 450 bases long and included the miRNA and its flanking sequence. Subsequent detection of the signals was done similar to mRNA Northern blotting with a few modifications. For detection of pre-miRNA, the probe was hybridized with the membrane at 50°C overnight and then washed twice at 50°C for 15 min. For detection of mature miRNA, the membrane was hybridized with the probe at 30°C overnight and then washed twice at 40°C for 15 min.

### RT-PCR to detect the spliced junction

Transfection of pCAG-RFP-miRNAint vector into NF-1 cells was done as described above. Twenty-four hours after transfection, the RNA was extracted from the cells. The cDNA was synthesized using the SuperScript III CellsDirect cDNA Synthesis Kit (Invitrogen) according to the instructions provided by the manufacturer using the following primers: 5'-GCTGGACATCACCTCCCACAACG-3' (forward) and 5'-ACAGGAGGTGGGGAGCAGGAGA-3' (reverse). The spliced intron would produce a PCR product of 212 bps while the unspliced intron would produce a PCR product of 701 bps. Platinum Taq DNA polymerase (Invitrogen) was used for PCR. The samples were denatured at 94°C for 2 minutes, followed by 35 cycles of (1) denaturation at 94°C for 30 seconds, (2) annealing at 64°C for 30 seconds and (3) extension at 72°C for 60 seconds. The PCR product was fractionated by electrophoresis on a 2% agarose gel. The amplified fragment was further purified by PCR purification kit (Qiagen) for sequencing analysis with the two primers.

### KGDHC activity assay

The activity assay for KGDHC was adapted from Shi et al. [54]. Three days after transfection with pCAG-RFP-miR-E1-1int, pCAG-RFP-miR-E2-4int, or pCAG-RFP-miR-Scrint, NF-1 cells in 6-well plates were washed twice with D-PBS buffer (Gibco). Total cell lysates were obtained after adding 250 μl/well of the lysate buffer followed by sonication and centrifgation. The lysate buffer contains 50 mM Tris-HCl (pH 8.0), 1 mM Dithiothreitol (DTT), 0.2 mM K_2_EGTA, 0.4% Triton X-100, and 50 μM leupeptin (all chemicals were obtained from Sigma unless indicated otherwise). Forty μl of the supernatant was added to the 160 μl activity assay buffer which consists of 63 mM Tris-HCl (pH8.0), 0.63 mM K_2_EDTA, 1.25 mM MgCl_2_, 1.25 mM CaCl_2_, 0.63 mM dithiothreitol (DTT), 0.4 mM thiamine pyrophosphate (TPP), 3.1 mM nicotinamide adenine dinucleotide (NAD), 0.4 mM coenzyme A, 0.13% Triton X-100, and 0.63 mM 2-ME. After 5 minutes incubation at 30°C, 10 μl of 0.1 M α-ketoglutarate was added to the reaction. The change of absorbance at A340 nm was recorded every minute for 18 minutes at 30°C with Tecan Safire microplate reader and the rates of the change were determined. The percentages of the rate relative to control were calculated.

### Statistical analysis

Results were presented as mean ± SE. Data were analyzed by Student's t-test. Differences were considered statistically significant if p < 0.05.

## Authors' contributions

LQ and ZX designed the constructs. LQ engineered and constructed the vectors and experimentally tested them. HW helped in Northern blots and in experimental design. XGX and HZ provided the Drosha shRNA plasmid and the BSENU6 vector. ZX and LQ wrote the manuscript. All authors have read and approved the final manuscript.
